# Loss of neuropeptide signalling alters temporal expression of mouse suprachiasmatic neuronal state and excitability

**DOI:** 10.1111/ejn.16590

**Published:** 2024-11-17

**Authors:** Sven Wegner, Mino D. C. Belle, Pi‐Shan Chang, Alun T. L. Hughes, Alexandra E. Conibear, Charlotte Muir, Rayna E. Samuels, Hugh D. Piggins

**Affiliations:** ^1^ Faculty of Biology, Medicine, and Health University of Manchester Manchester UK; ^2^ School of Physiology, Pharmacology, and Neuroscience University of Bristol Bristol UK; ^3^ School of Biological and Environmental Science Liverpool John Moores University Liverpool UK

**Keywords:** AMPA, circadian, gating, neurophysiology, SCN, sodium current, vasoactive intestinal polypeptide, VPAC_2_ receptor

## Abstract

Individual neurons of the hypothalamic suprachiasmatic nuclei (SCN) contain an intracellular molecular clock that drives these neurons to exhibit day‐night variation in excitability. The neuropeptide vasoactive intestinal polypeptide (VIP) and its cognate receptor, VPAC_2_, are synthesized by SCN neurons and this intercellular VIP‐VPAC_2_ receptor signal facilitates coordination of SCN neuronal activity and timekeeping. How the loss of VPAC_2_ receptor signalling affects the electrophysiological properties and states of SCN neurons as well as their responses to excitatory inputs is unclear. Here we used patch‐clamp electrophysiology and made recordings of SCN neurons in brain slices prepared from transgenic animals that do not express VPAC_2_ receptors (*Vipr2* ^*−/−*^ mice) as well as animals that do (*Vipr2* ^*+/+*^ mice). We report that while *Vipr2* ^*+/+*^ neurons exhibit coordinated day‐night variation in their electrical state, *Vipr2* ^*−/−*^ neurons lack this and instead manifest a range of states during both day and night. Further, at the population level, *Vipr2* ^*+/+*^ neurons vary the membrane threshold potential at which they start to fire action potentials from day to night, while *Vipr2* ^*−/−*^ neurons do not. We provide evidence that *Vipr2* ^*−/−*^ neurons lack a component of voltage‐gated sodium currents that contribute to SCN neuronal excitability. Moreover, we determine that this aberrant temporal control of neuronal state and excitability alters neuronal responses to a neurochemical mimic of the light‐input pathway to the SCN. These results highlight the critical role VIP‐VPAC_2_ receptor signalling plays in the temporal expression of individual neuronal states as well as appropriate ensemble activity and input gating of the SCN neural network.

## Graphical abstract

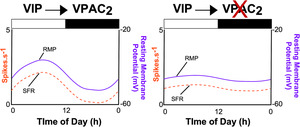

**Abbreviations**
aCSF: artificial cerebrospinal fluid
AMPA: α‐amino‐3‐hydroxy‐5‐methyl‐4‐isoxazolepropionic acid
AP: Action Potential
CC: Current clamp
DLAMOs: Depolarized low amplitude membrane oscillations
NMDA: N‐methyl‐D‐Aspartate
Per1: Period 1
RHT: Retinohypothalamic tract
SCN: Suprachiasmatic nuclei
SFR: Spontaneous firing rate
VC: Voltage clamp
VIP: Vasoactive intestinal polypeptide
*Vipr2 ^−/−^*
: Vasoactive intestinal polypeptide receptor 2 knock out
VPAC_2_
: Vasoactive intestinal polypeptide receptor 2
ZT: Zeitgeber Time

## 1 INTRODUCTION

From insects to mammals, electrical activity and intercellular signalling play pivotal roles in synchronizing the cellular elements that serve as endogenous daily or circadian pacemakers (Belle & Diekman, 2018; Hastings et al., 2018; Mohawk & Takahashi, 2011; Taghert & Nitabach, 2012). Such processes can enable these neural networks to shape the ensemble's collective response to synaptic inputs (Womelsdorf et al., 2014). This is exemplified in the mammalian brain's main circadian pacemaker located in the suprachiasmatic nuclei (SCN) (Curtis & Piggins, 2024). Environmental light information is conveyed directly to the SCN by the glutamatergic retinohypothalamic tract (RHT) and the nocturnal rodent SCN clock gates its response to this photic input pathway so that it can only be reset by light during the subjective night (Albrecht, 2012; Brown, 2016). How this gating is achieved is unclear, but one possibility is that the intracellular molecular clock in SCN neurons prepares their electrical states such that they manifest higher sensitivity to excitatory inputs at night than during the day (Colwell, 2011). Additionally, intrinsic intercellular signals that function to coordinate the timekeeping activities of SCN neurons may contribute to this gating by facilitating synchrony of neuronal states across the SCN network (Herzog, 2007).

One approach for gaining insight into the relationship of neuronal state and photic gating is through the investigation of a dysfunctional SCN network. The neuropeptide vasoactive intestinal polypeptide (VIP) and its cognate receptor, VPAC_2_, are made by SCN neurons (Kalamatianos et al., 2004; Todd et al., 2020) and in the absence of VIP‐VPAC_2_ receptor signalling, coordination among SCN cell‐autonomous clocks is compromised (Aton & Herzog, 2005; Hughes et al., 2008, 2021; Maywood et al., 2006; Patton et al., 2020) and in vivo photic gating is aberrant (Dragich et al., 2010; Hughes et al., 2004; Hughes & Piggins, 2008). Surprisingly, very little is known about how the loss of VIP signalling affects the properties and states of SCN neurons or their gating to excitatory inputs.

Since the SCN network is viable and readily accessible ex vivo (Green & Gillette, 1982; Groos & Hendriks, 1982; Shibata & Moore, 1988), we assessed neuronal state and gating to excitatory inputs by comparing whole‐cell current clamp and voltage‐clamp recordings from SCN‐containing brain slices prepared from animals with genetically targeted deletion of the VPAC_2_ receptor, *Vipr2* ^*−/−*^ mice (Harmar et al., 2002), as well as mice (*Vipr2* ^*+/+*^) that possess a fully functional SCN network. We report that at the population level, *Vipr2* ^*+/+*^ mouse neurons are depolarized during the day and hyperpolarized at night, while *Vipr2* ^*−/−*^ SCN neurons lack this day‐night change in membrane potential and instead manifest a broader range of states in membrane potential during day and night. Further, we show for the first time that the threshold in membrane voltage at which action potentials (APs) are discharged is significantly lower at night than during the day for *Vipr2* ^*+/+*^ SCN neurons, but such temporal variation is absent in *Vipr2* ^*−/−*^ SCN neurons. We provide evidence that components of voltage‐ and TTX‐sensitive sodium currents are a potential contributor to this day‐night variation in the *Vipr2* ^*+/+*^ AP firing threshold and that these currents are diminished or absent in *Vipr2* ^*−/−*^ neurons. Finally, we reveal, using a glutamatergic mimic of the light input pathway, that *Vipr2* ^*−/−*^ SCN neurons show reduced sensitivity at night, whereas *Vipr2* ^*+/+*^ neurons show similar sensitivity at day and night. Thus, intercellular neuropeptide signalling contributes to temporal phasing in the expression of SCN neuronal states, thereby shaping network excitability and gating to the light‐input pathway.

## 2 MATERIAL AND METHODS

### 2.1 Animal housing

All experiments were performed in accordance with the UK Animals (Scientific Procedures) Act of 1986 using procedures approved by The University of Manchester. Age‐matched adult (>8 weeks of age) male mice including those with intact neuropeptide signalling (*Vipr2* ^*+/+*^; n = 23) as well as mice lacking VPAC_2_ expression (*Vipr2* ^*−/−*^; n = 27) were used (see below). Animals were group housed under a 12 h light/12 h dark (LD) cycle. In LD conditions, lights‐on was defined as Zeitgeber time 0 (ZT0) and lights‐off as ZT12. Food (Bekay, B&K Universal, Hull, UK) and water were available ad libitum, the temperature was maintained at 20 ± 2°C and humidity at ~40%. Light intensity at the cage level in the breeding colony was ~45μWcm^−2^. The original breeding stock of *Vipr2* ^*−/−*^ animals was kindly provided by the late Prof. Tony Harmar, University of Edinburgh (see (Harmar et al., 2002) for details on the creation of this mouse line). Breeding stock for the *mPer1*::d2EGFP expressing or *Per1*::eGFP) mice was a gift from Prof. Douglas McMahon, Vanderbilt University (see (Kuhlman et al., 2000) for details on the creation of these mice). To generate *Vipr2* ^*−/−*^ mice in which *Per1* gene expression could be visualized at single cell level in brain slices, *Vipr2* ^−/−^ mice were crossed with *mPer1*::d2EGFP (*Per1*::GFP) expressing mice. Subsequently, animals homozygous for the disrupted *Vipr2* ^−/−^ transgene (*Vipr2* ^−/−^  ×  *Per1*::GFP; referred to as *Vipr2* ^−/−^) or homozygous for non‐disrupted, *Vipr2* gene (*Vipr2* ^*+/+*^  ×  *Per1*::GFP strain; referred to as *Vipr2* ^*+/+*^) were used.

### 2.2 Wheel‐running behaviour

To assess daily locomotor rhythms, *Vipr2* ^*−/−*^ and *Vipr2* ^*+/+*^ animals were singly housed in running wheel‐equipped cages with food and water available ad libitum. Rotation of the wheel was recorded using the acquisition suite within Chronobiology Kit (Stanford Software Systems, Santa Cruz, CA., USA). Mice were initially maintained under a 12 h light:12 h dark light–dark (LD) cycle for 2–3 weeks and then transferred into a constant dark (DD). To assess potential genotype differences in entrainment to the LD cycle, two measures were used: 1) the portion of wheel‐running occurring during the lights‐on and lights‐off phases (as extracted using the Chronobiology Kit) and 2) the time of activity onset on the first day following transfer to DD conditions as manually assessed by an experienced observer.

### 2.3 Preparation of brain slices

Mice were taken from the vivarium either early (ZT1–4; daytime recordings) or late in the day (ZT9–11; night‐time recordings) and deeply anaesthetized with isoflurane (Abbott Laboratories, Kent, UK) prior to cervical dislocation. Following removal, brains were placed in a chilled (4°C) incubation artificial cerebrospinal fluid (aCSF) containing in mM: NaCl 95; KCl 1.8; KH_2_PO_4_ 1.2; MgSO_4_ 7; NaHCO_3_ 26; Glucose 15; Sucrose 50; CaCl_2_ 0.5; phenol red 0.0014, pre‐gassed with 95% O_2_ and 5% CO_2_. Coronal slices (250 μm) were cut with a Campden 7000smz vibrating microtome (Campden Instruments Ltd., Leicestershire, UK). Slices were maintained in carbogen‐gassed aCSF at room temperature in a custom‐made brain slice keeper for 1–4 h prior to transfer to the recording chamber. Only slices corresponding to the intermediate level of the rostrocaudal axis of the SCN were used. Typically, one to two slices were used per mouse, with one to six neurons recorded per slice.

### 2.4 Electrophysiology recordings

Slices containing the mid‐coronal SCN section were incubated for at least 1 h in pre‐gassed recording aCSF (containing in mM: NaCl 127; KCl 1.8; KH_2_PO_4_ 1.2; MgSO_4_ 1.3; NaHCO_3_ 26; Glucose 15; CaCl_2_ 2.4; phenol red 0.0014) at room temperature within the recording chamber mounted on the stage of an Olympus BX51WI upright microscope (Olympus Corporation, Tokyo, Japan). The aCSF flow rate was 2–3 ml/min for all experiments. The microscope was equipped with inbuilt infrared differential contrast optics mounted on a vibration‐free air table (TMC, MA, USA). A Hitatchi C106005 CCD camera system was used for visualization of the cells on a high‐resolution black/white cathode ray monitor (Hitatchi Ltd., Tokyo, Japan). A Hamamatsu Orca R^2^ CCD camera system (Hamamatsu Photonics, Hamamatsu City, Japan) was used in combination with a X‐Cite 120 Q fluorescence light source (Excelitas, Waltham, MA, USA) for visualizing and discriminating the *Per1*:EGFP+ve from *Per1*::EGFP‐ve neurons. Unfortunately, the *Per1*‐driven signal was reduced in SCN slices from *Vipr2* ^*−/−*^ mice with few EGFP+ve cells per slice and consequently for analysis, we did not distinguish between EGFP+ve and ‐ve neurons and instead for both genotypes, we combined the data from these recordings. For daytime, recordings were made from ZT4 to ZT11.5, while for night‐time, recordings were made from ZT13 to 21. Photographs of the patch pipette and the puff pipette were taken in situ for accurate anatomical documentation of the recorded neurons within the SCN. Current‐clamp (CC) recordings were acquired as described previously (Belle et al., 2009, 2014; Timothy et al., 2018; Wegner et al., 2017). Briefly, current‐clamp (CC) recordings were done with a npi BA‐03X bridge amplifier (npi electronic GmbH, Tamm, Germany) or an Axoclamp 2A amplifier (Axon Instruments/Molecular Devices, Sunnyvale, USA) through a CED 1401 mk II A/D interface controlled by Spike2 software (Cambridge Electronic Design, Cambridge, UK). In pilot studies, no differences in the recorded parameters of SCN neurons were observed between the two amplifiers.

Voltage Clamp (VC) recordings were obtained with an Axopatch 200B amplifier (Molecular Devices) using methods adapted from (Jackson et al., 2004). Voltage‐activated ionic currents were evoked with fast depolarizing voltage steps (250 ms in 10 mV increments) applied from −80 to +40 mV from a holding potential of −70 mV. Patch pipettes (7–10 MΩ for CC recordings, 5 MΩ for VC recordings to reduce pipette capacitance) were pulled from thick‐walled borosilicate glass capillaries (GC150F‐10, Harvard Apparatus LTD, Kent, UK). Pipettes were filled with intracellular solution containing in mM: K‐Glutamate 130, KCl 10, MgCl2 2, Hepes 10, EGTA 0.5, K2ATP 2, NaGTP 0.5. All recordings were made at ~23°C. Access resistance was typically ~15 MΩ and series resistance (R_S_) ~ 20 MΩ, with R_S_ optimally compensated in all VC recordings. Neurons were omitted from analysis if the series resistance value during the recording changed by more than 15%.

### 2.5 Drugs and drug applications

All drugs were purchased from Tocris bioscience, UK. For stock solutions of α‐amino‐3‐hydroxy‐5‐methyl‐4‐isoxazolepropionic acid (AMPA) hydrobromide and tetrodotoxin (TTX) citrate powder was dissolved in distilled water. The working dilution for these drugs was prepared on the day of the experiment in pre‐gassed aCSF using established protocols (Belle et al., 2014; Itri et al., 2004). TTX was delivered by gravity‐controlled bath application. AMPA was delivered by an eight‐channel valve‐controlled pressurized perfusion system (ALA‐VM8, BPS‐8) through a QMM Quartz MicroManifold (ALA Scientific Instruments, New York, USA).

### 2.6 Data analysis

Data from CC recordings were analysed with Spike2 software (versions 6 and 7, Cambridge Electronic Design, Cambridge, UK) and the spontaneous firing rate (SFR) and the resting membrane potential (RMP) of the cells were measured. Both parameters were determined within 2–5 min establishing the whole‐cell configuration, and prior to investigations of the current injection. The RMP of these cells was determined by phase–plot averages using a previously described algorithm (Belle et al., 2009). Cells were removed from analysis if the recorded RMP was unstable, e.g., sudden, unaccountable changes of >3 mV or alterations in input resistance >15%. Only cells that were discharging APs or silent because of hyperpolarization were included for SFR analysis. Cells in depolarized states including those exhibiting depolarized low amplitude membrane oscillations (DLAMOs) were not used in SFR measurements. To measure the membrane threshold at which neurons initiated or terminated the firing of APs, slow depolarizing and hyperpolarizing current ramps were performed. Briefly spontaneously firing cells were silenced by a ~ 5–10 sec injection of ramping hyperpolarizing current so that the cell no longer fires followed by a depolarizing current ramp (~ 15 sec) to reinitiate firing. This membrane potential at which the neuron began firing again was interpreted as the AP firing threshold.

VC I‐V curves were analysed in Clampfit10.3 (Molecular Devices). For all statistical comparisons peak inward currents at either −20 mV or −10 mV holding potential were used (Jackson et al., 2004).

### 2.7 Statistics

Detailed statistical analyses were conducted using GraphPad Prism 10 (GraphPad Software, Boston, MA, USA), Kaleidagraph 5.0 (Synergy Software, Reading, PA, USA) and SPSS (Statistical Product and Service Solutions, IBM) as well as on‐line resources (www.vassarstats.net; www.estimationstats.com). Fisher's exact test (www.vassarstats.net) was employed to assess genotype and time of day differences in the proportions of SCN neurons in different states. To evaluate genotype and time of day effects on the properties of SCN neurons, the normal distribution of the data was assessed using the Kolmogorov–Smirnov test with normally distributed data subsequently analysed using two‐way analysis of variance (ANOVA) and post hoc pairwise comparison tests (Bonferroni corrected). In cases where the data did not exhibit a normal distribution, generalized linear model (GLM)‐ Type III tests followed by Bonferroni post hoc tests were used. To estimate the effect size in the ANOVA and GLM analysis, we used paired differences within the estimation statistics framework (www.estimationstats.com) (Ho et al., 2019). Estimation statistics report mean differences (effect size) as well as with confidence interval estimates to express uncertainty. This methodology generates a bootstrap sampling distribution for each paired mean difference, employing 5000 bootstrap samples, and then bias‐correcting and accelerating the confidence intervals.

For most experimental components in which the same neurons were challenged with different doses of drug, genotype and dose effects were evaluated using two‐way repeated measures ANOVA with post hoc Bonferroni or Tukey tests. To generate dose–response relationships, doses were log10 transformed and fitted with the Hill slope constrained to 2 (Graphpad Prism). T‐tests (Kaleidagraph) were also used to test group differences in tetrodotoxin studies. For one experiment using a VPAC_2_ antagonist, we used independent t‐tests. For all statistical analyses, the threshold for significance was p < 0.05. A detailed breakdown of the statistical analysis is reported in Supplemental Table S1.

Graphs were made using Kaleidagraph or Graphpad Prism or via online (www.estimationstats.com). Figures were constructed using Inkscape (www.inkscape.org/.) and Canvas X Draw (Canvas GFX, Inc., Boston, MA, USA). In figures, data are plotted either as jitter plot, as mean ± SEM or as mean with fitted distribution and mean ± 95% confidence intervals (estimation plots).

## 3 RESULTS

### 3.1 Daily wheel‐running and temporal control of SCN neuronal state are comprised by loss of VIP‐VPAC_2_ receptor signalling

**FIGURE 1:**
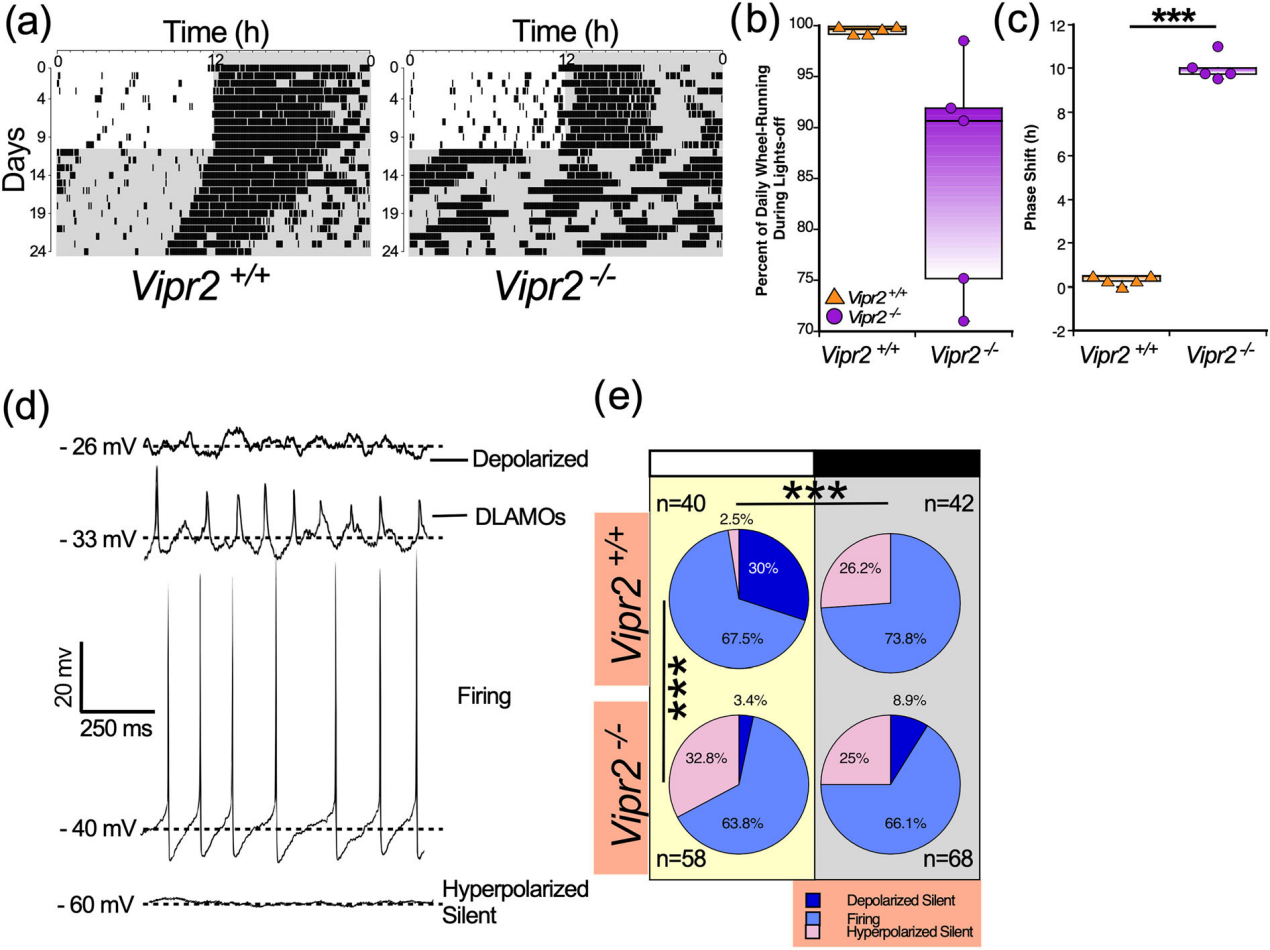
*Vipr2* ^*−/−*^ mice exhibit altered photic entrainment and lack daily change in the expression of SCN neuronal state. Representative wheel‐running actograms of a *Vipr2* ^*+/+*^ (left) and a *Vipr2* ^*−/−*^ mouse (right) illustrate that under light–dark conditions, both exhibit similar synchronization to lights‐off (a), with most activity confined to the dark phase (b). However, when transferred to constant dark, *Vipr2* ^*+/+*^ initiate wheel‐running in close alignment with the projected time of lights‐ off, while *Vipr2* ^*−/−*^ animals unmask and become active 9‐10 h prior to this projected time of lights‐off, indicating that photic entrainment is significantly altered in *Vipr2* ^*−/−*^ mice (c). In current clamp mode, *Vipr2* ^*+/+*^ and *Vipr2* ^*−/−*^ SCN neurons exhibit a range of states (d). In the neurochemically intact *Vipr2* ^*+/+*^ SCN but not the *Vipr2* ^*−/−*^ SCN, the expression of neuronal state varies from day to night (e). SCN neuronal state differs between *Vipr2* ^*+/+*^ and *Vipr2* ^*−/−*^ during the day. ***p < 0.005.

Under 12 h–12 h LD conditions, both *Vipr2* ^*+/+*^ and *Vipr2* ^*−/−*^ mice showed apparent entrainment, confining the majority (99.6 ± 0.17% and 85.5 ± 5.3%, respectively) of their daily wheel‐running activity to the lights‐off phase (Figure 1a, b; *Vipr2* ^*+/+*^ vs *Vipr2* ^*−/−*^; t = 2.67, df = 4, p = 0.055). On release into DD, clear and significant genotype differences emerged. In the first 24 h period in DD, *Vipr2* ^*−/−*^ mice initiated vigorous sustained wheel‐running ~9–10 h in advance of the projected time of lights‐off, whereas *Vipr2* ^*+/+*^ animals began wheel‐running ~0.5 h prior to the projected time of lights‐off (Figure 1a,c; *Vipr2* ^*+/+*^ vs *Vipr2* ^*−/−*^; t = 34.84, df = 4, p < 0.001). These observations are consistent with other studies (Aton et al., 2005; Brown et al., 2005; Colwell et al., 2003; Hannibal et al., 2011; Harmar et al., 2002; Hughes et al., 2004; Hughes & Piggins, 2008) and illustrate that under LD conditions, VIP‐signalling deficient mice exhibit only apparent entrainment.

In SCN brain slices prepared from either genotype, we attempted to use epifluorescence to distinguish neurons in which *Per1*‐driven green fluorescent protein could be visualized (EGFP+ve neurons) from those in which it could not (EGFP‐ve neurons). However, in the *Vipr2* ^*−/−*^ SCN, the *Per1*‐driven EGFP signal was diminished with few cells detectable per slice and consequently we combined data from EGFP‐ve and EGFP+ve neurons. To investigate how the electrical state and membrane properties of *Vipr2* ^*+/+*^ SCN neurons vary across the projected LD cycle and to determine if and how these properties are altered in *Vipr2* ^*−/−*^ mice, we made whole‐cell current clamp recordings from SCN brain slices. Using previously published protocols (Belle et al., 2009, 2014; Timothy et al., 2018; Wegner et al., 2017) we classified the spontaneous activity of SCN cells into one of three states: 1) depolarized and not discharging action potentials (silent) or with DLAMOs, 2) spontaneously discharging action potentials (APs) and 3) hyperpolarized and silent (Figure 1d). Circadian clock gene expression is damped in the *Vipr2* ^*−/−*^ SCN in vivo (Harmar et al., 2002; Hitrec et al., 2023), and consistent with previous reports (Hughes et al., 2008; Maywood et al., 2006).

All states were recorded from *Vipr2* ^*+/+*^ (n = 82 cells from 11 animals) and *Vipr2* ^*−/−*^ SCN (n = 116 cells from 14 animals) neurons and in *Vipr2* ^*+/+*^ but not *Vipr2* ^*−/−*^ SCN brain slices, the proportion of cells in the different states was associated with time of day (Fisher's exact test, p = 0.00004 (for *Vipr2* ^*+/+*^ and p > 0.05 [p = 0.382] for *Vipr2* ^*−/−*^ slices; Figure 1e). In the *Vipr2* ^*+/+*^ SCN, depolarized cells (silent and DLAMO states) collectively constituted ~30% of neurons recorded during the day, while at night, they were not detected. Neurons in the hyperpolarized and silent state increased from ~2.5% during the day to ~26% at night. By contrast, in *Vipr2* ^*−/−*^ brain slices, depolarized states (silent or expressing DLAMOs) were infrequent in the day (~3.4% of cells) and night (~8.9% of cells) phases, while those recorded in the hyperpolarized silent state represented ~33% of cells recorded during the day and ~25% of those recorded at night (Figure 1). During the day, SCN cell state differed from *Vipr2* ^*+/+*^ to *Vipr2* ^*−/−*^ mice (p = 4 × 10^−7^), while at night, there was no genotype difference (p = 0.155). Thus, day‐night change in the spontaneous state of SCN neurons is altered in the *Vipr2* ^*−/−*^ SCN, with *Vipr2* ^*−/−*^ neurons expressing a distribution in neuronal states during day and night that resembles that expressed at night in the *Vipr2* ^*+/+*^ SCN (see Supplemental Table S1 for more detailed statistical analysis).

### 3.2 Daily variation in membrane potential and spontaneous firing rate is absent in the *Vipr2* ^*−/−*^ SCN

**FIGURE 2:**
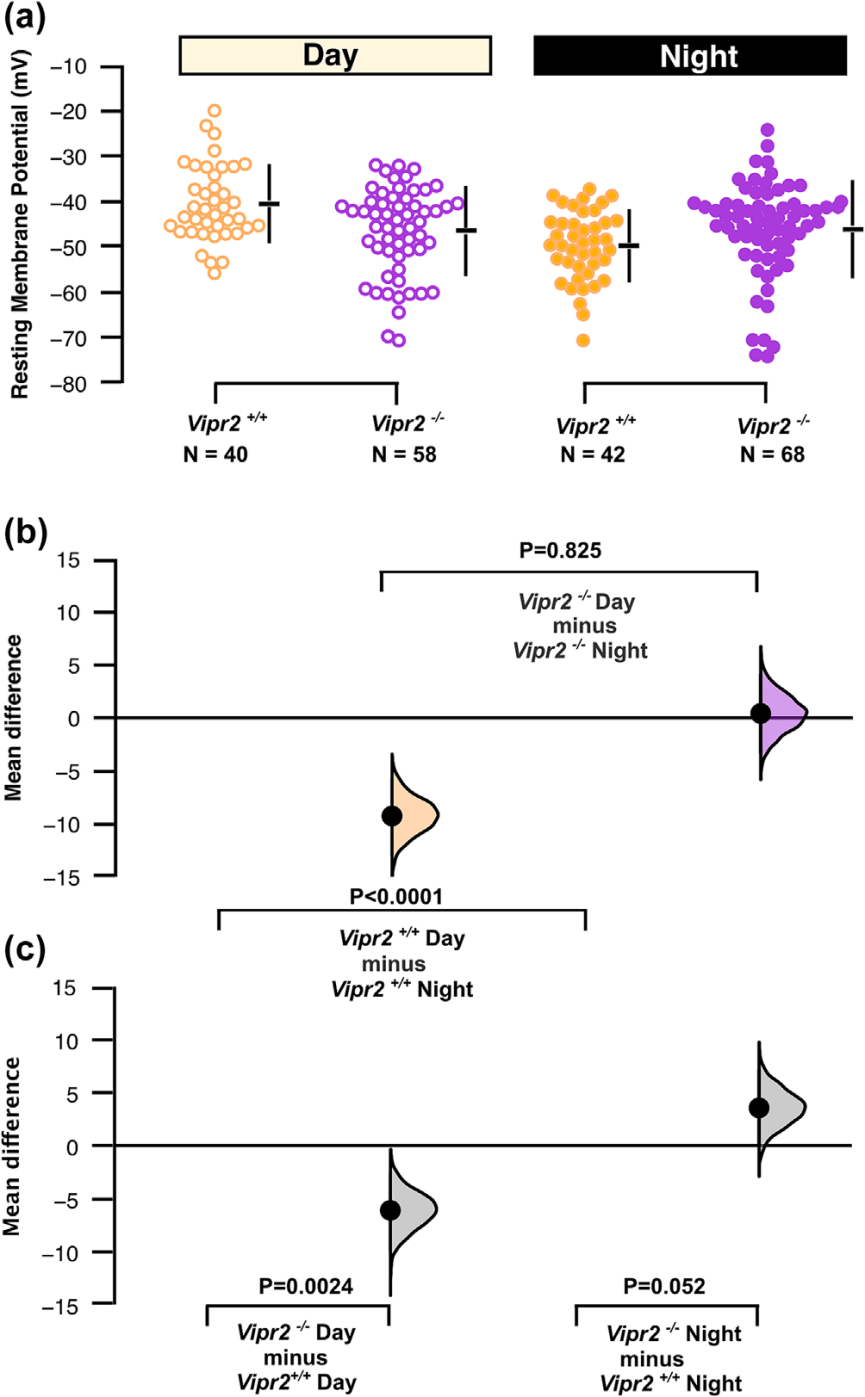
Effect of genotype and time of day on membrane potential of suprachiasmatic neurons. The mean difference for four comparisons is shown in the Cumming estimation plots (b and c). The raw data are plotted by genotype and time of day in (a) with 95% confidence intervals are indicated by the ends of the vertical error bars and the mean value indicated for each genotype/time of day by the horizontal bar. In (b and c), each mean difference is plotted as a bootstrap sampling distribution with mean differences are depicted as the filled larger black dots.

**FIGURE 3:**
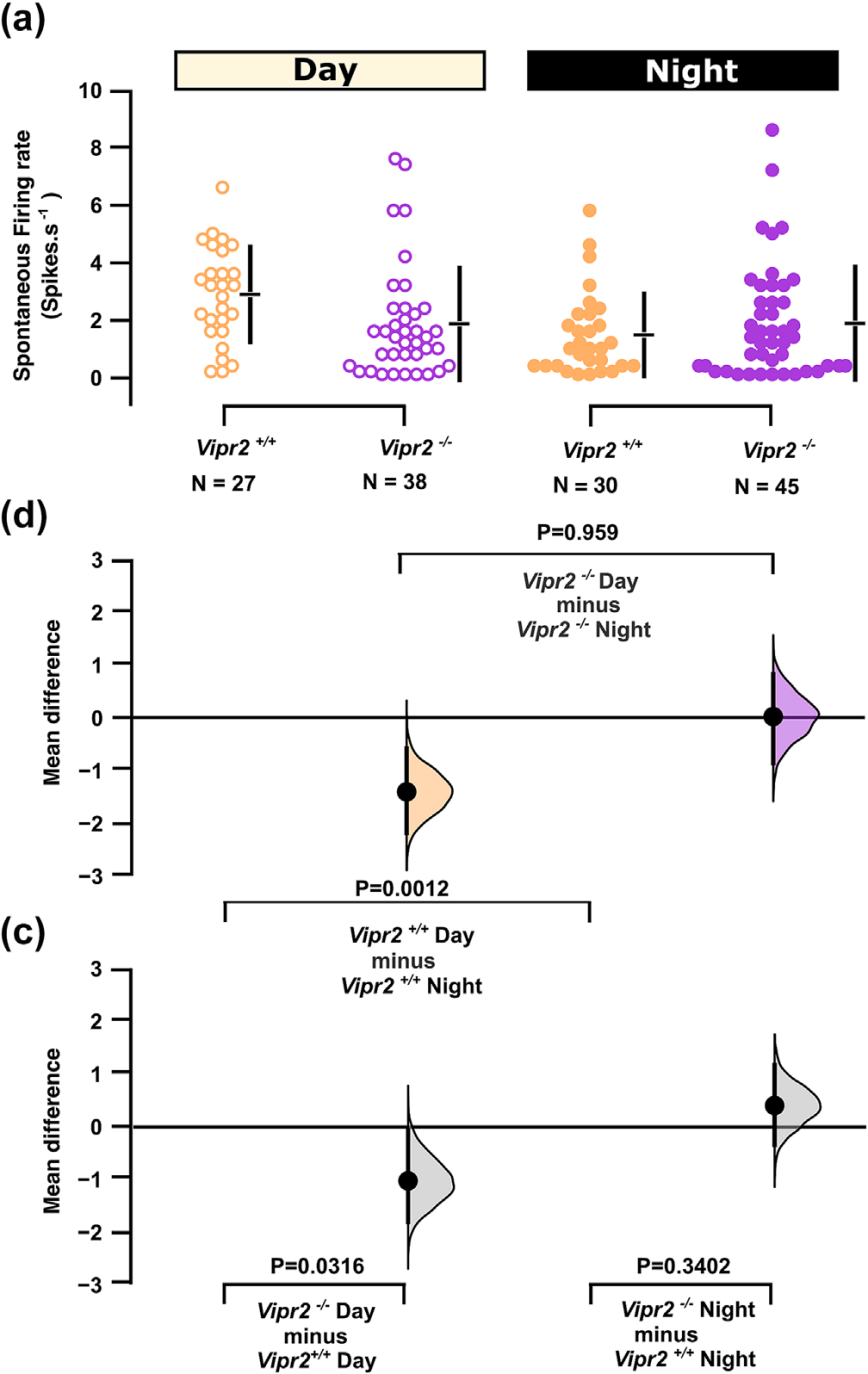
Effect of genotype and time of day on spontaneous firing rate of suprachiasmatic neurons. The mean difference for four comparisons is shown in the Cumming estimation plots (b and c). The raw data are plotted by genotype and time of day in (a) with 95% confidence intervals indicated by the ends of the vertical error bars and the mean value indicated for each genotype/time of day by the horizontal bar. In (b and c), each mean difference is plotted as a bootstrap sampling distribution with mean differences depicted as the filled larger black dots.

This temporal difference in the timing of neuronal state is also reflected in the resting membrane potential (RMP, Figure 2) and the spontaneous firing rate (SFR, Figure 3) of recorded SCN cells. For RMP, two‐way ANOVA showed a significant main effect of time of day (F_1,165_ = 11.37, p < 0.001) as well as time of day x genotype interaction (F_1_=13.63, p < 0.001) with RMP varying from day to night in *Vipr2* ^*+/+*^ (−42.8 ± 1.5 vs − 49.2 ± 1.3 mV; mean ± SEM; pairwise comparison, p = 0.017; with an effect size as measured by permutation t‐test, p < 0.0001), but not *Vipr2* ^*−/−*^ SCN neurons (−44.3 ± 1.3 mV vs − 48.2 ± 1.3 mV; pairwise comparison, p > 0.05; permutation t‐test, p = 0.825) (Figure 2a,c). RMP varied between *Vipr2* ^*−/−*^ and *Vipr2* ^*+/+*^ neurons during day (permutation t‐test, p = 0.0024) or night (permutation t‐test, p = 0.052; Figure 2e). Both time of day (Wald c^2^, p = 0.016) and the time of day x genotype interaction (Wald c^2^, p = 0.013) were factors for SFR which differed from day to night in *Vipr2* ^*+/+*^ neurons (2.14 ± 0.23vs 1.4 ± 0.28 Hz; pairwise comparison, p = 0.015; permutation t‐test, p = 0.0012), but not *Vipr2* ^*−/−*^ neurons (1.93 ± 0.3 vs 1.88 ± 0.29 Hz; p > 0.05, permutation t‐test, p = 0.96) (Figure 3a,c). Permutation t‐tests further indicated a significant effect size for genotype during the day (p = 0.032; SFR of *Vipr2* ^*−/−*^ neurons lower than that of *Vipr2* ^*+/+*^ neurons), but not the night (p = 0.34; Figures 3e) (see Supplemental Table S1 for more detailed statistical analysis). These findings are broadly consistent with a previous extracellular recording study of *Vipr2* ^*−/−*^ mouse SCN neurons (Cutler et al., 2003) and provide evidence that at the ensemble level, *Vipr2* ^*−/−*^ SCN neurons do not vary in membrane potential or spontaneous firing rate from day to night.

### 3.3 Time of day modulation of response to a glutamatergic signal is compromised by loss of the VPAC_2_ receptor

A cardinal property of circadian clocks is their ability to control if, when, and how they respond to environmental signals. As illustrated above and in other studies (Colwell et al., 2003; Hannibal et al., 2011; Harmar et al., 2002; Hitrec et al., 2023; Hughes et al., 2021; Hughes & Piggins, 2008) disruption of VIP‐VPAC_2_ receptor signalling results in abnormal entrainment of mice to the LD cycle as well as impairing encoding of daylength (Lucassen et al., 2012). Further, *Vipr2* ^*−/−*^ mice show aberrant responses to acute photic stimuli: 15 min pulses of light can immediately activate intracellular transduction pathways in the *Vipr2* ^*−/−*^ SCN at any time of the circadian cycle, whereas activation of these pathways by light is mostly restricted to the night in the SCN of *Vipr2* ^*+/+*^ mice (Hughes et al., 2004). Since the main photic input to the SCN is glutamatergic, we next tested if such photic gating may arise from temporal regulation of individual SCN neurons by neurochemically mimicking the photic input through brief (3 s) pulses of the glutamatergic agonist AMPA to SCN brain slices (Ang et al., 2021; Mizoro et al., 2010). Further, we evaluated how the loss of the VPAC_2_ receptor influenced the response of SCN neurons to this neurochemical proxy of the light input pathway.

**FIGURE 4:**
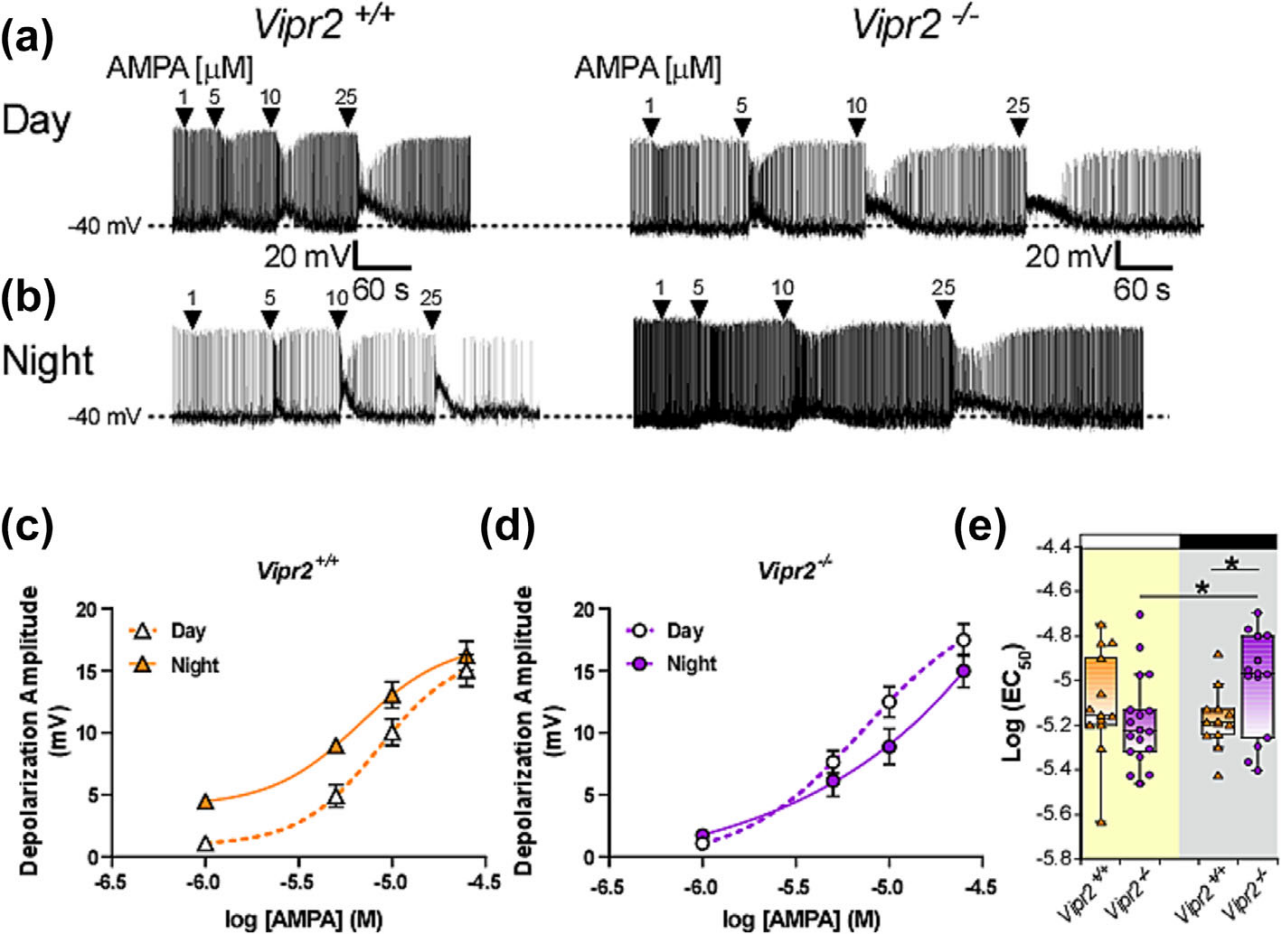
Nocturnal sensitivity to a glutamatergic input to SCN neurons is altered in the *Vipr2* ^*−/−*^ SCN neurons. Representative current‐clamp recordings from *Vipr2* ^*+/+*^ and *Vipr2* ^*−/−*^ SCN neurons illustrate depolarizing responses to pulses of AMPA (1–25 μM) assessed during day (a) and night (b) epochs. Plots of the amplitude of the depolarization response to AMPA against the log concentration of AMPA illustrate increased nocturnal response of *Vipr2* ^*+/+*^ SCN neurons at low concentrations (c) not seen in *Vipr2* ^*−/−*^ neurons recorded at night (d). Symbols in (c) and (d) represent the mean ± SEM, while solid and broken lines represent the fitted function. Comparison of the Log(EC_50_) of AMPA calculated from the plots in (c) and (d) indicate that it is increased from day to night in *Vipr2* ^*−/−*^ SCN neurons and is higher than in *Vipr2* ^*+/+*^ neurons at this time. Symbols in box plots represent values individual neurons, with horizontal black line representing the median value. *p < 0.05.

We evaluated if the amplitude of responses to AMPA of current‐clamp recorded neurons in the ventral SCN (vSCN; the region of the SCN receiving substantial retinal innervation (Abrahamson & Moore, 2001; Antle & Silver, 2005)) differed from day to night as well as with the loss of VPAC_2_ receptor signalling. To minimize the influence of RMP and to make recordings comparable between genotypes, all cells were manually held at a membrane potential of ~ − 40 mV and the effects of increasing concentrations of AMPA (1, 5, 10 and 25 μM) tested on *Vipr2* ^*+/+*^ and *Vipr2* ^*−/−*^ vSCN neurons recorded during the day or night (Figure 4a,b).

Depolarization in membrane potential in response to AMPA treatment was recorded from both *Vipr2* ^*+/+*^ and *Vipr2* ^*−/−*^ SCN neurons (Figure 4a,b). AMPA dose‐relatedly depolarized membrane potential of *Vipr2* ^*+/+*^ neurons during the day (n = 13 cells in slices from 3 animals) and night (n = 13 cells in slices from 3 animals), with larger responses recorded during the night [Dose: F_1.8, 43.09_ = 131.3, p < 0.001; Time of Day F_1,30_ = 8.48, p = 0.008; Dose x Time of Day interaction: F_1.795, 43.09_ = 1.596]. For *Vipr2* ^*−/−*^ mice, dose‐related depolarizing responses to AMPA were observed during day (n = 18 cells in slices from 3 animals) and night (n = 14 cells in slices from 3 animals) [Dose: F_1.8, 53.65_ = 169.36 (p < 0.001) and Dose x Time of Day interaction: F_1.8,53.65_ = 4.23 (p = 0.023)]. To further examine the relationships in the effects of AMPA on SCN neuronal excitability dose–response curves (Hill slope constrained to = 2) were fitted (Figure 4c,d) and Log(EC_50_) and Emax were calculated. The Log(EC_50_) differed from day to night in *Vipr2* ^*−/−*^ (t = −2.295, df = 30, p = 0.029; Figure 4e; permutation t‐test, p = 0.029; Supplemental Figure S1), but not *Vipr2* ^*+/+*^ mice (Figure 4e; Supplemental Figure S1). Further, at night, the Log(EC_50_) of *Vipr2* ^*+/+*^ SCN neurons varied from that of *Vipr2* ^*−/−*^ SCN neurons (t = 2.25, df = 25, p = 0.033, permutation t‐test, p = 0.029; Supplemental Figure S1). By contrast, Emax did not differ between *Vipr2* ^*+/+*^ and *Vipr2* ^*−/−*^ mice nor from day to night in either genotype (Supplemental Figure S2) (see Supplemental Table S1 for more detailed statistical analysis). Collectively, these parametric and curve‐fitting analyses provide evidence that genetic perturbation of VIP‐VPAC_2_ receptor signalling enhances nocturnal responsiveness to lower concentrations of a neurochemical mimic of the light input pathway to the SCN.

### 3.4 Components of TTX‐sensitive sodium currents are altered in *Vipr2* ^*−/−*^ neurons

**FIGURE 5:**
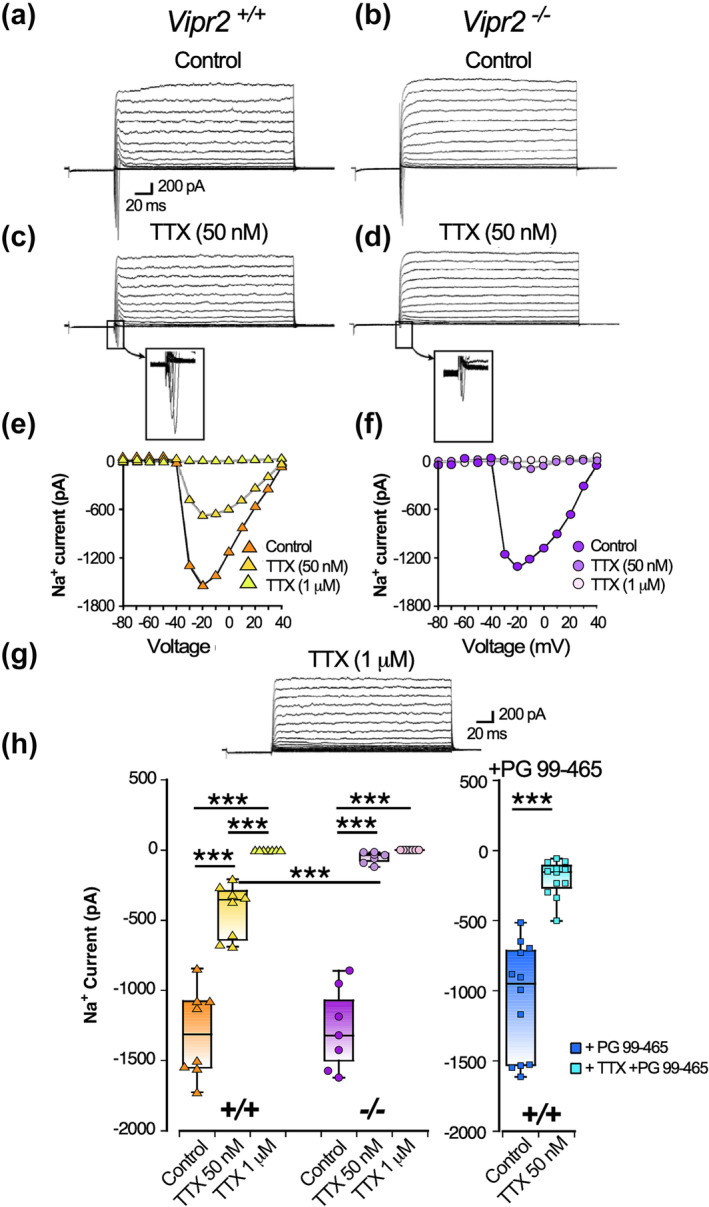
*Vipr2* ^*−/−*^ neurons lack tetrodotoxin (TTX)‐resistant sodium current(s). Voltage clamp recordings of representative *Vipr2* ^*+/+*^ (a) and *Vipr2* ^*−/−*^ (b) neurons illustrate that while *Vipr2* ^*+/+*^ neurons exhibit currents that are sustained in the presence of TTX (50 nM; rectangle (c) and its expanded view) such currents are abolished by 50 nM TTX in *Vipr2* ^*−/−*^ SCN neurons (d; rectangle and its expanded view). Current vs voltage plots in (e) and (f) show that evoked currents are sustained in 50 nM in *Vipr2* ^*+/+*^ but not *Vipr2* ^*−/−*^ neurons and that these currents are abolished by higher TTX concentration (1 μM) in *Vipr2* ^*+/+*^ neurons (e) as illustrated in the voltage clamp recording in (g). Suppression of sodium currents in *Vipr2* ^*+/+*^ and *Vipr2* ^*−/−*^ neurons by TTX (h) with 50 nM TTX abolishing currents in *Vipr2* ^*−/−*^ but not *Vipr2* ^*+/+*^ neurons. In the presence of the VPAC_2_ receptor antagonist, PG99–465, *Vipr2* ^*+/+*^ SCN neurons exhibited lack of resistance to the suppression of sodium currents by 50&amp; nM of TTX. In (h) symbols represent sodium current measurement of individual neurons, while horizontal black line in box plots illustrates the corresponding median value. ***p&amp; <&amp; 0.005.

The above experiments indicate that as a population, neurons in the *Vipr2* ^*−/−*^ deficient SCN do not exhibit the typical daily variations neuronal state, membrane potential, spontaneous firing rate as well as responsiveness to the light input pathway that are observed in the neurochemically intact SCN. To gain insight into how intrinsic excitability is altered by the loss of the VPAC_2_ receptor, we assessed sodium currents since these contribute to the spontaneous AP firing of rodent SCN neurons (Bean, 2007; Jackson et al., 2004; Pennartz et al., 1997). We initially sought to determine if components of tetrodotoxin (TTX)‐sensitive sodium currents varied between the genotypes. To do this, we made voltage‐clamp recordings and measured how TTX (50 nM and 1 μM) influenced the amplitude of sodium currents in *Vipr2* ^*+/+*^ and *Vipr2* ^*−/−*^ SCN neurons at night (n = 8 cells from slices prepared from 3 animals of each genotype; Figure 5a‐g). We used these concentrations as pilot studies of SCN neurons recorded in current‐clamp mode indicated that *Vipr2* ^*−/−*^ neurons were more responsive than *Vipr2* ^*+/+*^ neurons to low (50 nM) as well as higher (1 μM) concentrations of TTX (see Figure 7). Neurons were randomly sampled in ventral SCN. Both TTX dose [F_1.26, 16.385_ = 258.186, p < 0.001] and genotype x TTX treatment interaction [F_1.26,16.385_ = 6.092, p = 0.019] were factors on the amplitude of sodium currents_,_ while the effect of genotype was not [F_1,13_ = 3.522, p = 0.083]. Under control conditions, currents of similar amplitude were evoked by the step‐wise protocol in both genotype, but treatment with the submaximal dose (50 nM) of TTX revealed a component of sodium current in *Vipr2* ^*+/+*^ SCN cells that was significantly attenuated in *Vipr2* ^*−/−*^ SCN neurons (pairwise comparison, p < 0.001) (Figure 5c‐f)). Subsequently, application of 1 μM TTX completely abolished any residual sodium currents in both genotype, an observation that is consistent with previous work in SCN neurons (Kononenko et al., 2004; Pennartz et al., 1997) (Figure 5 e‐h). Since *Vipr2* ^*−/−*^ mice develop in the absence of the VIP‐VPAC_2_ receptor signalling, we next tested whether blockade of VPAC_2_ receptors in the adult *Vipr2* ^*+/+*^ SCN also alters sensitivity to TTX (50 nM). Application of the VPAC_2_ antagonist, PG 99–465, did not alter the amplitude of evoked sodium current (control: ~1300 pA vs control + PG 99–465–1064 pA; t‐test, p = 0.17; n = 13 cells), but did increase sensitivity to 50 nM TTX (TTX alone ~ − 433 pA vs PG 99–465 + TTX ~ 200 pA; t‐test, p = 0.004) (Figure 5h). Thus pharmacological blockade of VPAC_2_ receptors in the adult *Vipr2* ^*+/+*^ SCN can recapitulate some aspects of the lack of TTX‐resistance of *Vipr2* ^*−/−*^ SCN neurons (see Supplemental Table S1 for more detailed statistical analysis). This suggests that effects arising from developing in the absence of VIP‐VPAC_2_ signalling cannot solely explain this altered sensitivity to TTX of *Vipr2* ^*−/−*^ SCN neurons (but see also (Mazuski et al., 2020)).

**FIGURE 6:**
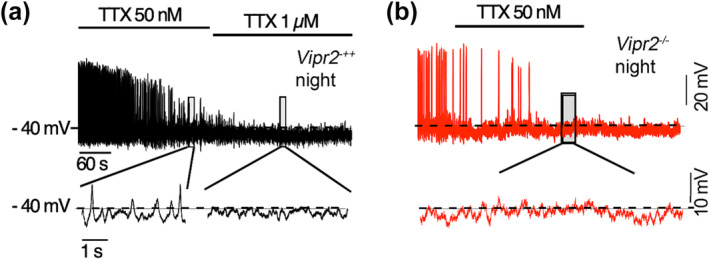
Depolarizing oscillations in membrane potential of *Vipr2* ^*−/−*^ but not *Vipr2* ^*+/+*^ neurons are abolished by a low concentration of TTX. Current clamp recordings of representative *Vipr2* ^*+/+*^ (black; a) and *VIpr2* ^*−/−*^ neurons (red; b) at night reveal that low amplitude oscillations in membrane potential are sustained with application of tetrodotoxin (TTX; 50 nM) in Vipr2^+/+^ (6/6 neurons), but not *Vipr2* ^*−/−*^ neurons (3/8 neurons) tested (expanded recording traces in lower row correspond to recording epoch within the shaded rectangles in the upper traces). These membrane oscillations are lost with application of 1 μM) TTX.

The above voltage‐clamp recordings provide evidence that *Vipr2* ^*−/−*^ neurons lack important TTX‐resistant currents and we next examined how bath‐applied TTX (50 nM and 1 μM) affected AP firing and membrane events in *Vipr2* ^*+/+*^ and *Vipr2* ^*−/−*^ SCN neurons recorded in current clamp mode. With 5 min application of 50 nM TTX, AP firing in *Vipr2* ^*+/+*^ neurons was gradually eliminated to reveal small fluctuations in membrane potential that persisted in 50 nM TTX (6 of 6 cells tested from 3 animals), but were subsequently abolished by treatment with 1 μM TTX (Figure 6a). Such sodium current(s) in *Vipr2* ^*+/+*^ SCN cells may provide subthreshold depolarization drives enabling the membrane to reach threshold to fire APs. A similar time‐course of treatment with 50 nM TTX silenced AP firing of *Vipr2* ^*−/−*^ neurons and abolished the small fluctuations of membrane potential seen under these conditions in *Vipr2* ^*+/+*^ neurons (Figure 6b; 5 of 8 cells from 3 animals; significant genotype difference: Fishers Exact test p = 0.031) (see Supplemental Table S1 for more detailed statistical analysis). These findings support the view that *Vipr2* ^*−/−*^ SCN neurons lack some TTX‐resistant sodium current(s) observed in *Vipr2* ^*+/+*^ neurons and provide evidence that they are deficient in an intrinsic excitatory mechanism.

### 3.5 Day‐night variation in AP firing threshold is lost in the *Vipr2* ^*−/−*^ SCN

**FIGURE 7:**
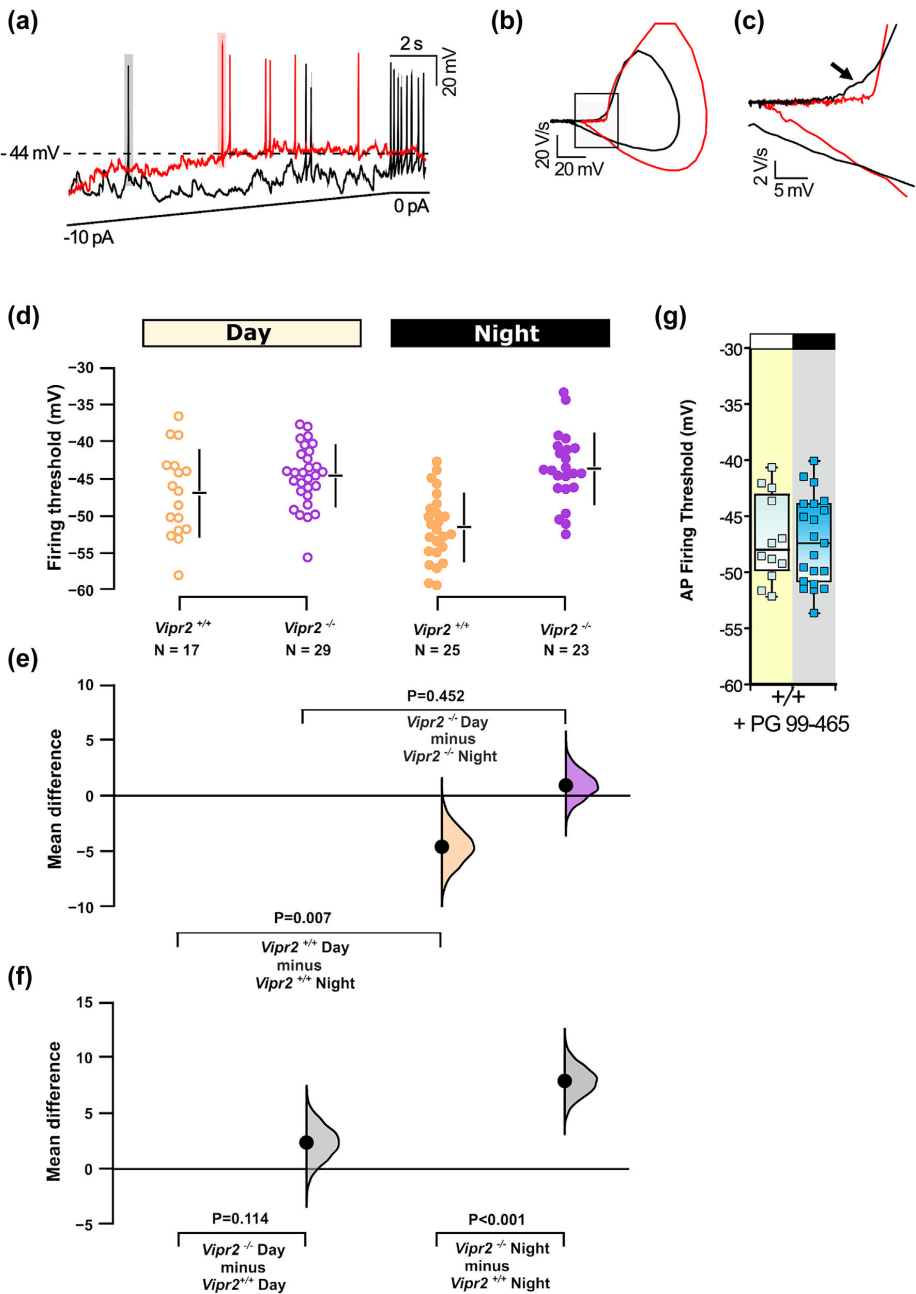
Firing threshold is reduced at night in *Vipr2* ^*+/+*^ but not *Vipr2* ^*−/−*^ SCN neurons. Representative current‐clamp recordings of a *Vipr2* ^*+/+*^ (black) and a *Vipr2* ^*−/−*^ (red) neuron (a). With manual injection of current ramp (to 10 pA), the *Vipr2* ^*+/+*^ neuron initiates firing (spike highlighted with grey shading) at a lower threshold than the *Vipr2* ^*−/−*^ neuron (spike highlighted with light red shading). Phase‐plane plot reveals that a deflection of voltage observed in the initiation of the spike in *Vipr2* ^*+/+*^ neurons is absent in *Vipr2* ^*−/−*^ neurons [black rectangle in (b) which is enlarged in (c). The raw data are plotted by genotype and time of day in (d), with 95% confidence intervals indicated by the ends of the vertical error bars and the mean value indicated for each genotype/time of day by the horizontal bar. The mean difference for eight comparisons are shown in the Cumming estimation plots (e, f). In (e, f), each mean difference is plotted as a bootstrap sampling distribution with mean differences are depicted as the filled larger black dots. The firing threshold is reduced at night for *Vipr2* ^*+/+*^ but not *Vipr2* ^*−/−*^ neurons (f). For *Vipr2* ^*−/−*^ neurons, the firing threshold at night is more depolarized than that of corresponding *Vipr2* ^*+/+*^ neurons (f). Treatment of *Vipr2* ^*+/+*^ neurons with the VPAC_2_ antagonist, PG99–465 abolishes this day‐night difference in firing threshold (g). In (g), symbols represent firing rate threshold of individual neurons, while horizontal black line in box plots illustrates the corresponding median value.

We next sought to determine if and how the membrane potential at which AP firing is initiated differs from day to night and between the genotypes. To assess this, we made targeted recordings from neurons randomly distributed within the SCN of both genotypes. Neurons were recorded in current clamp mode and subjected to a current ramp to determine the membrane potential at which the cells either ceased or initiated AP firing (Figure 7a). We also use phase‐plots to reveal any underlying depolarizing subthreshold events that contribute to driving AP firing (Figure 7b,c). Two‐way analysis of variance indicated a significant main effect of genotype (F_1,90_ = 27; p < 0.001), and a significant genotype x time of day interaction (F_1,90_ = 7.74; p = 0.007). The AP firing threshold of *Vipr2* ^*+/+*^ SCN neurons during the day (−47 ± 1.2 mV,

n = 17 cells) differed to that recorded at night (−51.6 ± 0.9 mV, n = 25; pairwise comparison, p = 0.003; permutation t‐test, p = 0.007; Figure 7d,f), whereas AP firing threshold measured in *Vipr2* ^*−/−*^ cells did not vary from day (−44.6 ± 0.9 mV, n = 29) to the night (−43.7 ± 1 mV, n = 23; pairwise comparison, p > 0.05; permutation t‐test, p > 0.05; Figure 7d). At night, the AP firing threshold of *Vipr2* ^*−/−*^ neurons occurred at a significantly more depolarized level compared to that measured in *Vipr2* ^*+/+*^ neurons at this time (pairwise comparison, p < 0.001; permutation t‐test, p < 0.001; Figure 7h) (see Supplemental Table S1 for more detailed statistical analysis). These analyses show that with the loss of VIP‐VPAC_2_ receptor signalling, SCN neurons at night require a more depolarized membrane potential to fire APs.

Collectively, these studies provide evidence that genetically targeted impairment of VIP‐VPAC_2_ receptor signalling compromises the daily control of the membrane potential and the firing of action potentials.

Next, we investigated if pharmacological blockade of VPAC_2_ receptors in adult *Vipr2* ^*+/+*^ mice would also alter the AP threshold of SCN neurons. Previously, we showed that short‐term treatment with the VPAC_2_ receptor antagonist, PG99–465, for 5–10 min prior to VIP application, acutely antagonizes VIP's presynaptic effects in the SCN (Pakhotin et al., 2006), while the longer‐term application (>3 h) results in alteration of the cell's intrinsic electrophysiological behaviour (Cutler et al., 2003; Itri & Colwell, 2003). Therefore, for 4 h prior to recording, SCN‐containing brain slices from *Vipr2* ^*+/+*^ mice were incubated in aCSF containing PG99–465. We found that with this longer‐term VPAC_2_ receptor blockade with PG99–465, there was no day‐night difference in the AP firing threshold (−47 ± 1.12 mV, n = 12 cells from 3 animals vs − 47.6 ± 1.04 mV, n = 17 cells from 3 animals; t‐test, p > 0.05; Figure 7j) (see Supplemental Table S1 for more detailed statistical analysis). Thus, pharmacological blockade of VIP‐VPAC_2_ signalling in *Vipr2* ^*+/+*^ cells mimicked the AP firing threshold recorded at night in *Vipr2* ^*−/−*^ SCN neurons.

## 4 DISCUSSION

From drosophila to mammals, intercellular neuropeptide signalling is established as playing critical roles in coordinating the excitability and coupling of neuronal circadian clock cells (Glossop, 2011; Hastings et al., 2019; Kunst et al., 2015; Mohawk & Takahashi, 2011). Nonetheless, how the loss of neuropeptide signals influences clock neuron excitability and gating to synaptic inputs has remained under‐explored. Here, we show for the first time that at the population level, the absence of the VPAC_2_ receptor abolishes day‐night variation in the expression of resting state and membrane potential of SCN neurons with daily variation in spontaneous firing rate also suppressed. The absence of the VPAC_2_ receptor compromises cellular gating to a neurochemical mimic of the light input pathway. Further, we find in the *Vipr2* ^*+/+*^ mouse SCN that a probable contributor to this gating is a nocturnal reduction in the membrane threshold for firing action potentials. This is absent through the loss of functional VIP‐VPAC_2_ receptor signalling which alters this day‐night variation in the action potential firing threshold. We then provide evidence that at night, *Vipr2* ^*−/−*^ SCN neurons lack TTX‐resistant sodium currents which potentially contributes to their inability to gate excitatory stimuli. These findings extend our knowledge of neuropeptide signalling in circadian circuits to highlight their importance in a diverse range of clock neuronal states as well as gating this neural network's response to an important environmental resetting stimulus.

Our demonstration that readily detectable day‐night variation in SCN neuronal excitability is abolished in *Vipr2* ^*−/−*^ mice reveals for the first time how the targeted loss of this G‐protein coupled receptor influences neuronal membrane properties in clock circuits. Previous in vitro research conducted on adult mouse SCN neurons restricted to the day phase, demonstrated a tendency for *Vipr2* ^*−/−*^ SCN neurons to be more hyperpolarized than *Vipr2* ^*+/+*^ SCN cells (Pakhotin et al., 2006). Further, in extracellular recordings made from adult brain slices in vitro, many *Vipr2* ^*−/−*^ SCN neurons do not vary the rate of spontaneously discharged APs from day to night (Brown et al., 2005; Cutler et al., 2003). The current findings demonstrate that adult *Vipr2* ^*−/−*^ SCN neurons maintain a moderate level of RMP and SFR across both day and night. This also reflects the damped level of clock gene expression in the SCN over the circadian cycle of *Vipr2* ^*−/−*^ mice in vivo and in vitro (Harmar et al., 2002; Hitrec et al., 2023; Hughes et al., 2008, 2015).

Several ion currents are implicated in regulating SCN neuronal activity and function (Belle & Diekman, 2018; Brown & Piggins, 2007; Colwell, 2011; Harvey et al., 2020). We found that a potential contributing mechanism to the absence of temporal expression of neuronal state in the *Vipr2* ^*−/−*^ mouse SCN is the partial loss of sodium currents (Figures 5, 6). Components of these currents in *Vipr2* ^*+/+*^ neurons at night are resistant to 50 nM TTX, but were completely blocked by 1 μM TTX (Figure 5). These currents also enable the *Vipr2* ^*+/+*^ cells to fire APs at relatively low membrane potential (~mean of −51 mV) (Figure 7). This current is absent in the majority of *Vipr2* ^*−/−*^ SCN neurons tested such that 50 nM TTX completely suppresses depolarizing oscillations in their membrane potential. Since these cells initiate the firing of APs at a similarly depolarized level (~mean of −45 mV) day or night (Figure 7) and have a higher Log(EC_50_) to AMPA at night than during the day or in comparison to *Vipr2* ^*+/+*^ cells at night (Figure 4), then this argues for both loss of an intrinsic mechanism of excitability as well as temporal partitioning of excitability in *Vipr2* ^*−/−*^ neurons. Long‐term incubation of adult *Vipr2* ^*+/+*^ neurons with the VPAC_2_ receptor antagonist, PG99–465, can re‐capitulate some of these observations, indicating that these effects are unlikely to be solely attributable to the developmental artefact. Sodium channel subunits expressed in the SCN include Na_V_1.5, 1.8 and 1.9 (Colwell, 2011; Pennartz et al., 2002) but as we did not concomitantly block other ionic currents, further research is needed to fully identify and address the bases of these currents.

Data from neuroanatomical studies suggests that VIP‐VPAC_2_ receptor signalling is widespread in the SCN; most neurochemically‐defined cell populations express VPAC_2_ receptor mRNA (Kalamatianos et al., 2004; Kalló et al., 2004; King et al., 2003; Wen et al., 2020), and VIP binding sites (Morin et al., 1994) as well as VPAC_2_ protein are present throughout most of the SCN (An et al., 2013; Hughes et al., 2021). Consistent with this, we found that the loss of VIP‐VPAC_2_ receptor signalling altered excitability states of SCN neurons, with significant population‐level effects.

In general terms, the spontaneous firing rate of mouse SCN neurons decreases from a higher frequency of discharge during the middle of the day (ZT4–8) to low frequencies across the night (ZT12–22) (Albus et al., 2002; Brown et al., 2006; Cutler et al., 2003; Kuhlman & McMahon, 2004; Nakamura et al., 2008). Interestingly, recent studies targeting arginine vasopressin (AVP) synthesizing or VIP‐expressing SCN neurons find that at least subpopulations of these neurons increase firing rate over the middle (VIP neurons: ZT17–20, (Collins et al., 2020)) or late‐night phases (AVP: ZT21.5–24 (Gizowski et al., 2016)). In our study, we recorded nocturnal activity of neurons across a broad epoch (ZT14–21) and found that some neurons in the *Vipr2* ^*+/+*^ and *Vipr2* ^*−/−*^ SCN discharge at elevated rate (> 3 spikes/s) at night‐time. This suggests that AVP and VIP neurons were sampled in our assessment of the night‐time firing rate of SCN neurons. Since at the ensemble level, *Vipr2* ^*−/−*^ neurons do not vary membrane potential or firing rate from day to night, we infer that loss VIP‐VPAC_2_ receptor signalling broadly compromises the circadian drive on AP firing of SCN neurons.

As shown in this study as well as others (Colwell et al., 2003; Harmar et al., 2002; Hughes et al., 2004), animals with compromised VIP‐VPAC_2_ signalling show abnormal entrainment to the light–dark cycle. Here under light–dark conditions, we observed that the *Vipr2* ^*−/−*^ mice align their active phase to the lights‐off phase, but when transferred to constant dark, they show an immediate advance in the onset of their active phase. This is unlike the *Vipr2* ^*+/+*^ animals who begin their free‐run in broad alignment with the previous time of lights‐off. Thus, *Vipr2* ^*−/−*^ mice show ‘masking’ to light (they suppress active behaviour during lights‐on, but become active with the beginning of lights‐off; (Milićević et al., 2022)) but do not properly entrain to the light–dark cycle. Interestingly, we found that while SCN neurons of the *Vipr2* ^*+/+*^ show elevated depolarizing responses to AMPA at night, *Vipr2* ^*−/−*^ mice did not and expressed similar responses to AMPA day and night. Since glutamate released from the retinohypothalamic tract acts via AMPA and NMDA receptors to convey photic information to the SCN (Brown, 2016), this altered responsiveness to AMPA may contribute to the abnormal entrainment of *Vipr2* ^*−/−*^ mice to the light–dark cycle. Our observations that the AP firing threshold was reduced from day to night in the *Vipr2* ^*+/+*^ SCN but not the *Vipr2* ^*−/−*^ SCN and that the AP firing threshold of *Vipr2* ^*−/−*^ SCN neurons resembled that measured from the *Vipr2* ^*+/+*^ SCN during the day raises the possibility that there is general dysregulation in the generation of APs as well as responses to an excitatory input in the *Vipr2* ^*−/−*^ SCN. This is consistent with our previous in vivo investigation in which we reported that intracellular markers of neuronal activity (phosphorylated extracellular signal‐regulated kinase I/II and c‐Fos) varied from high levels during the middle of the day to low levels at night in the *Vipr2* ^*+/+*^ SCN but not in *Vipr2* ^*−/−*^ SCN where they were low both day and night. Further, while brief pulses of light elevated these markers only during the night in the *Vipr2* ^*+/+*^ SCN, light pulses elevated the expression of these markers in the *Vipr2* ^*−/−*^ SCN throughout the day and night (Hughes et al., 2004). Thus, both in vivo and in vitro, loss of VIP‐VPAC_2_ receptor signalling compromises ensemble SCN neuronal integration and processing of excitatory input.

A limitation of this study is that we have assessed the impact of the loss of VIP‐VPAC_2_ receptor signalling in male mice only and consequently we cannot comment on whether similar effects on SCN neuronal activity are seen in female mice. A second limitation is that we investigated sodium currents without simultaneously blocking other ionic conductances and thus we cannot rule out that such currents also contribute to the altered excitability of *Vipr2* ^*−/−*^ SCN neurons. Similarly, to be definitive, a larger sample of cells of each genotypes needs to be tested. A third limitation is that both NMDA and AMPA receptors are involved in mediating the actions of RHT activation by light on the SCN (Brown et al., 2006; Ebling, 1996) and as we tested AMPA only, potential alterations in NMDA activity in the *Vipr2* ^*−/−*^ SCN remain unknown. Similarly, the neuropeptide, pituitary adenylate cyclase‐activating polypeptide (PACAP), is also involved in RHT signalling to the SCN (Hannibal et al., 2017) and whether there are alterations in PACAP signalling in *Vipr2* ^*−/−*^ mice remains to be comprehensively assessed.

In summary, this study reveals for the first time that in the absence of the VPAC_2_ receptor, SCN neurons fail to exhibit day‐night variation in neuronal state, such that at the network level, they lack a daily rhythm in cellular RMP and SFR. We provide evidence that sodium currents that ordinarily contribute to time‐of‐day changes in the firing of action potentials are lost in the *Vipr2* ^*−/−*^ SCN network. Further, while *Vipr2* ^*+/+*^ neurons show enhanced responses to a glutamatergic mimic of the photic input during the circadian night, *Vipr2* ^*−/−*^ SCN neurons do not. Therefore, impairment of photic gating in the SCN of these mice is attributable to losses of temporal control of neural network as well as alteration in voltage‐gated sodium channel regulation of neuronal excitability.

## AUTHOR CONTRIBUTIONS

Sven Wegner performed and analysed current‐clamp whole‐cell recordings and wrote the initial draft of the manuscript. Mino D. C. Belle performed and analysed voltage‐clamp recordings and taught Sven Wegner patch‐clamp electrophysiology. Pi‐Shan Chang and Charlotte Muir assisted with statistical analysis and figure construction. Alun T. L. Hughes and Mino D. C. Belle advised on experimental design. Alexandra E. Conibear assisted with curve‐fitting and dose–response analysis. Rayna E. Samuels assisted with animal husbandry. Hugh D. Piggins supervised Sven Wegner and did additional statistical analysis, wrote and edited the submitted version of the manuscript. The authors dedicate this study to the memory of Prof. Steve Brown, explorer of mountains and the brain.

## CONFLICT OF INTEREST STATEMENT

None.

### PEER REVIEW

The peer review history for this article is available at https://www.webofscience.com/api/gateway/wos/peer-review/10.1111/ejn.16590.

## Supporting information


**Figure S1.** The half‐maximal effective concentration of AMPA differs between genotypes at night and between day and night in the *Vipr2−/−* SCN. The mean difference for eight comparisons is shown in the Cumming estimation plots (b) and (c). The raw data are plotted by genotype and time of day in (a) with 95% confidence intervals are indicated by the ends of the vertical error bars and the mean value indicated for each genotype/time of day by the horizontal bar. In (b) and (c), each mean difference is plotted as a bootstrap sampling distribution with mean differences are depicted as the filled larger black dots.
Figure S2 The maximum effect of AMPA does not differ between genotypes. The mean difference for eight comparisons is shown in the Cumming estimation plots (b) and (c). The raw data are plotted by genotype and time of day in (a) with 95% confidence intervals are indicated by the ends of the vertical error bars and the mean value indicated for each genotype/time of day by the horizontal bar. In (b) and (c), each mean difference is plotted as a bootstrap sampling distribution with mean differences are depicted as the filled larger black dots.

## Data Availability

The data that support the findings of this study are available from the corresponding author upon reasonable request.
