# Supplementary material for: Loss of neuropeptide signalling alters temporal expression of mouse suprachiasmatic neuronal state and excitability
Source: Eur J Neurosci. 2024 Nov 17;60(11):6617–33. doi: 10.1111/ejn.16590 (PMC11612845; doi:10.1111/ejn.16590)

Supplemental Table S1

Summary Statistics for Figures 1-7 and Figures S1 and 2.

Figure No.	Panel	n	Test used	Test value and DF	P value	Post-hoc test
Figure 1	b	$Vipr2^{+/+}$=5; $Vipr2^{-/-}$=5	Unpaired t test (2 tailed)	$t_{(4)}$=2.676	P=0.0553	
	c	$Vipr2^{+/+}$=5; $Vipr2^{-/-}$=5	Unpaired t test (2 tailed)	$t_{(4)}$=-34.8434	P<0.0001	
	e	Day:$Vipr2^{+/+}$=40; $Vipr2^{-/-}$=58<br>Night:$Vipr2^{+/+}$=42; $Vipr2^{-/-}$=68	Fisher's exact test		$Vipr2^{+/+}$: Day vs Night P<0.0001	
					$Vipr2^{-/-}$: Day vs Night P=0.3829	
					Day: $Vipr2^{+/+}$ vs $Vipr2^{-/-}$ P<0.0001	
					Night: $Vipr2^{+/+}$ vs $Vipr2^{-/-}$ P=0.1550	
Figure 2	b	Day:$Vipr2^{+/+}$=40; $Vipr2^{-/-}$=58	Estimation Stats: permutation test	95% CI:(-9.654)-(-2.588)	P=0.0024	
		Night:$Vipr2^{+/+}$=42; $Vipr2^{-/-}$=68	Estimation Stats: permutation test	95% CI:(0.184)-(7.140)	P=0.0520	
	c	Day:$Vipr2^{+/+}$=40; $Vipr2^{-/-}$=42	Estimation Stats: permutation test	95% CI:(-12.903)-(-5.891)	P<0.0001	
		Night:$Vipr2^{+/+}$=58; $Vipr2^{-/-}$=68	Estimation Stats: permutation test	95% CI:(-3.175)-(3.964)	P=0.8248	
Figure 3	b	Day:$Vipr2^{+/+}$=27; $Vipr2^{-/-}$=38	Estimation Stats: permutation test	95% CI:(-7.799)-(-0.062)	P=0.0316	
		Night:$Vipr2^{+/+}$=30; $Vipr2^{-/-}$=45	Estimation Stats: permutation test	95% CI:(-0.338)-(1.173)	P=0.3402	
	c	Day:$Vipr2^{+/+}$=27; $Vipr2^{-/-}$=30	Estimation Stats: permutation test	95% CI:(-2.193)-(-0.583)	P=0.0012	
		Night:$Vipr2^{+/+}$=38; $Vipr2^{-/-}$=45	Estimation Stats: permutation test	95% CI:(-0.866)-(0.823)	P=0.9590	
Figure 4	e	Day:$Vipr2^{+/+}$=13; $Vipr2^{-/-}$=18<br>Night:$Vipr2^{+/+}$=12; $Vipr2^{-/-}$=13	2 way ANOVA	Genotype:F (1, 56) = 0.000	P=1.0000	
				Day/Night:F (1, 56) = 0.000	P=1.0000	
				Interaction:F (1, 56) = 6.551	P=0.0130	$Vipr2^{+/+}$: Day vs Night P=0.017<br>Night:$Vipr2^{+/+}$ vs $Vipr2^{-/-}$ P=0.017
Figure 5	h	$Vipr2^{+/+}$=8<br>Vipr2-/-=8	Two-way RM ANOVA	Genotype:F (1, 14) = 0.7185	P=0.4109	
				Conc (1.132, 15.85) = 132.4	P<0.0001	$Vipr2^{+/+}$: Baseline vs 50 nM P=0.0005<br>$Vipr2^{+/+}$: Baseline vs 1 uM P=0.0005<br>$Vipr2^{+/+}$: 50 nM vs 1 uM P=0.0102<br>$Vipr2^{-/-}$: Baseline vs 50 nM P=<0.0001<br>$Vipr2^{-/-}$: Baseline vs 1 uM P=<0.0001<br>$Vipr2^{-/-}$: 50 nM vs 1 uM P=0.0011
				Interaction:F (2, 28) = 4.431	P=0.0213	50 nM TTX: Vipr2+/+ vs Vipr2-/- P=0.0023
		$Vipr2^{+/+}$ +PG Con=9;<br>$Vipr2^{+/+}$+PG+50 nM TTX=9	Unpaired t test (2 tailed)	$t_{(22)}$=7.103	P<0.0001	

Figure No.	Panel	n	Test used	Test value and DF	P value
Figure 7	e	$Vipr2^{+/+}$: day=17; night=25	Estimation Stats: permutation test	95% CI:(-7.795)-(-1.280)	P=0.0072
		$Vipr2^{-/-}$:day=29; night=23	Estimation Stats: permutation test	95% CI:(-1.349)-(3.461)	P=0.4524
	f	Day:$Vipr2^{+/+}$=17; $Vipr2^{-/-}$ =29	Estimation Stats: permutation test	95% CI:(-0.692)-(5.379)	P=0.1148
		Night:$Vipr2^{+/+}$=25; $Vipr2^{-/-}$ =23	Estimation Stats: permutation test	95% CI:(5.327)-(10.463)	P<0.0001
	g	$Vipr2^{+/+}$ +PG: Day=12; Night=21	Unpaired t test (2 tailed)	$t_{(23)}$=0.165	P=0.8695

S1	b	$Vipr2^{+/+}$: day=13; night=13	Estimation Stats: permutation test	95% CI:(-0.205)-(0.078)	P=0.3450
		$Vipr2^{-/-}$:day=18; night=14	Estimation Stats: permutation test	95% CI:(0.013)-(0.312)	P=0.0290
	c	Day:$Vipr2^{+/+}$=13; $Vipr2^{-/-}$ =18	Estimation Stats: permutation test	95% CI:(-0.229)-(0.081)	P=0.2780
		Night:$Vipr2^{+/+}$=13; $Vipr2^{-/-}$ =14	Estimation Stats: permutation test	95% CI:(0.173)-(0.289)	P=0.0292

S2	b	$Vipr2^{+/+}$: day=13; night=13	Estimation Stats: permutation test	95% CI:(-4.560)-(3.580)	P=0.8660
		$Vipr2^{-/-}$:day=18; night=14	Estimation Stats: permutation test	95% CI:(-5.630)-(3.580)	P=0.3110
	c	Day:$Vipr2^{+/+}$=13; $Vipr2^{-/-}$ =18	Estimation Stats: permutation test	95% CI:(-2.060)-(6.220)	P=0.9740
		Night:$Vipr2^{+/+}$=13; $Vipr2^{-/-}$ =14	Estimation Stats: permutation test	95% CI:(-3.350)-(3.550)	P=0.2020

Supplemental Figure Captions:

Supplemental Figure S1

The half-maximal effective concentration of AMPA differs between genotypes at night and between day and night in the *Vipr2*$^{-/-}$ SCN.

The mean difference for 8 comparisons are shown in the Cumming estimation plots (b) and (c). The raw data are plotted by genotype and time of day in (a) with 95% confidence intervals are indicated by the ends of the vertical error bars and the mean value indicated for each genotype/time of day by the horizontal bar. In (b) and (c), each mean difference is plotted as a bootstrap sampling distribution with mean differences are depicted as the filled larger black dots.

Supplemental Figure S2

The maximum effect of AMPA does not differ between genotypes.

The mean difference for 8 comparisons are shown in the Cumming estimation plots (b) and (c). The raw data are plotted by genotype and time of day in (a) with 95% confidence intervals are indicated by the ends of the vertical error bars and the mean value indicated for each genotype/time of day by the horizontal bar. In (b) and (c), each mean difference is plotted as a bootstrap sampling distribution with mean differences are depicted as the filled larger black dots.

Supplemental Figure S1

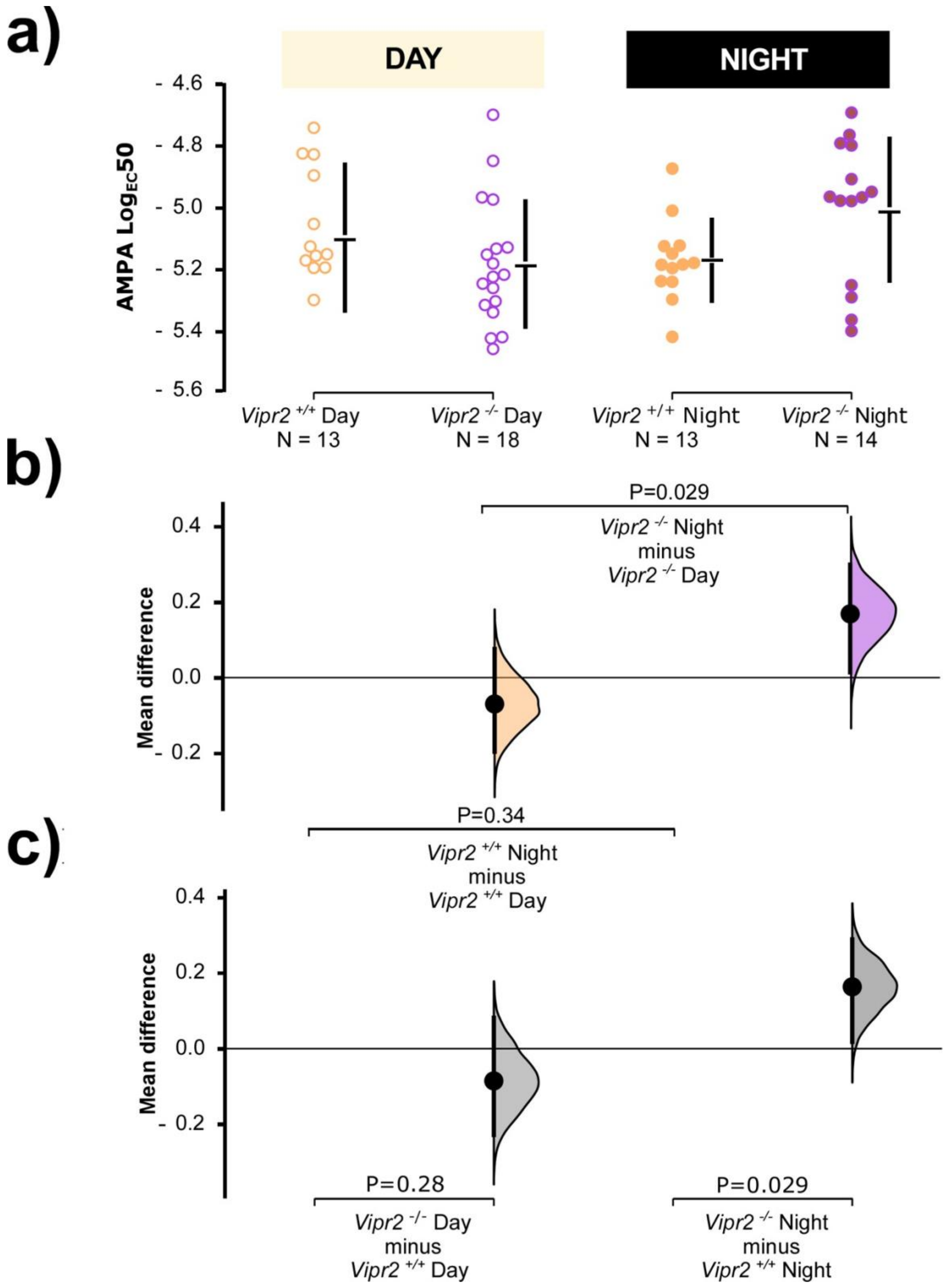

Supplemental Figure S2

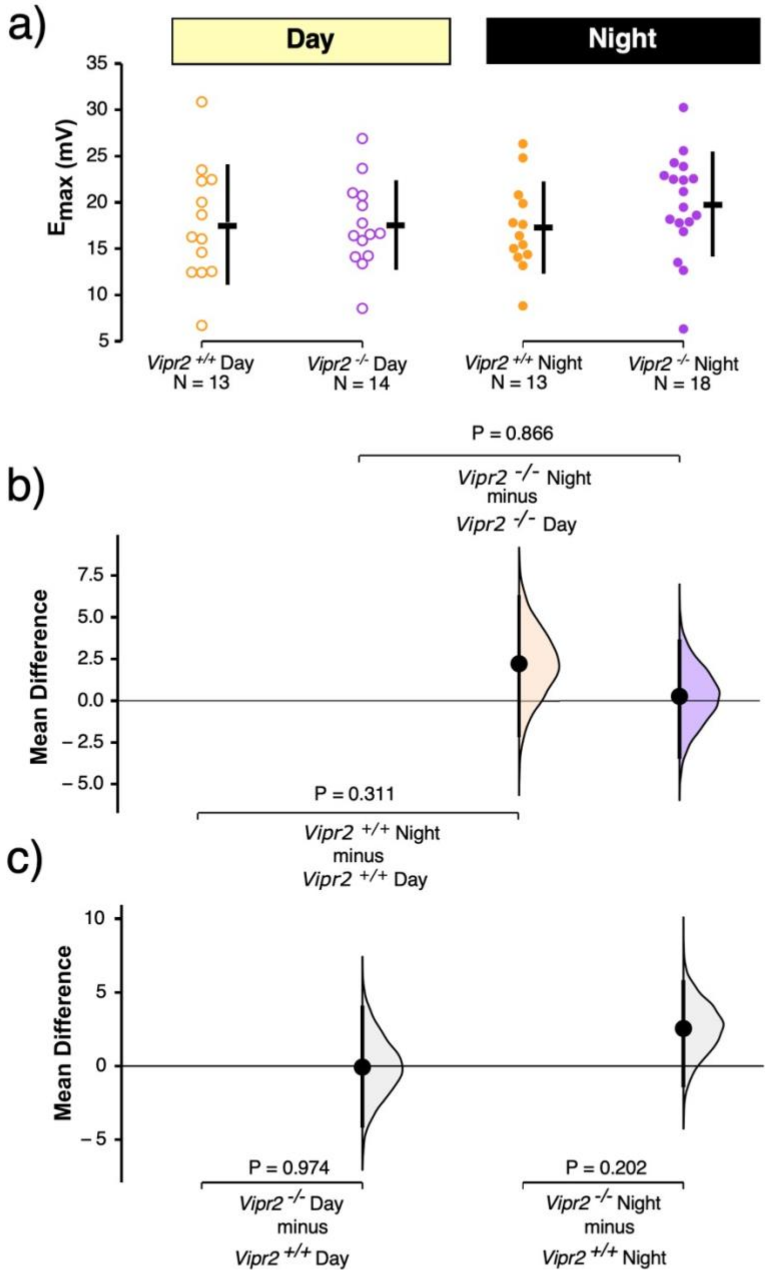

Supplement: Supplementary file 1 — Figure S1. The half‐maximal effective concentration of AMPA differs between genotypes at night and between day and night in the Vipr2−/− SCN. The mean difference for eight comparisons is shown in the Cumming estimation plots (b) and (c). The raw data are plotted by genotype and time of day in (a) with 95% confidence intervals are indicated by the ends of the vertical error bars and the mean value indicated for each genotype/time of day by the horizontal bar. In (b) and (c), each mean difference is plotted as a bootstrap sampling distribution with mean differences are depicted as the filled larger black dots. Figure S2 The maximum effect of AMPA does not differ between genotypes. The mean difference for eight comparisons is shown in the Cumming estimation plots (b) and (c). The raw data are plotted by genotype and time of day in (a) with 95% confidence intervals are indicated by the ends of the vertical error bars and the mean value indicated for each genotype/time of day by the horizontal bar. In (b) and (c), each mean difference is plotted as a bootstrap sampling distribution with mean differences are depicted as the filled larger black dots. [file EJN-60-6617-s001.pdf]
